# Synthesis and Structure-Activity Relationships of a Series of Aporphine Derivatives with Antiarrhythmic Activities and Acute Toxicity

**DOI:** 10.3390/molecules21121555

**Published:** 2016-11-28

**Authors:** Hui Wang, Xin Cheng, Shujun Kong, Zixian Yang, Hongmei Wang, Qiuyan Huang, Jingyu Li, Cheng Chen, Yunshu Ma

**Affiliations:** Department of Pharmaceutics Science, School of Chinese Materia Medica, Yunnan University of Traditional Chinese Medicine, 1076#, Yuhua road, Chenggong, Kunming 650000, China; huiwang2016@sina.cn (H.W.); chengxin920@126.com (X.C.); kongshujun1@126.com (S.K.); yangzixian2@126.com (Z.Y.); 15198906044@163.com (H.W.); 15912165160@163.com (Q.H.); lijingyu06@163.com (J.L.); guoke10141@163.com (C.C.)

**Keywords:** aporphine derivatives, antiarrhythmia, 10,11-dibromocrebanine, 3-bromocrebanine, crebanine, stephanine

## Abstract

Some aporphine alkaloids, such as crebanine, were found to present arrhythmic activity and also higher toxicity. A series of derivatives were synthesized by using three kinds of aporphine alkaloids (crebanine, isocorydine, and stephanine) as lead compounds. Chemical methods, including ring-opening reaction, bromination, methylation, acetylation, quaternization, and dehydrogenation, were adopted. Nineteen target derivatives were evaluated for their antiarrhythmic potential in the mouse model of ventricular fibrillation (VF), induced by CHCl_3_, and five of the derivatives were investigated further in the rat model of arrhythmia, induced by BaCl_2_. Meanwhile, preliminary structure-activity/toxicity relationship analyses were carried out. Significantly, *N*-acetamidesecocrebanine (**1d**), three bromo-substituted products of crebanine (**2a**, **2b**, **2c**), *N*-methylcrebanine (**2d**), and dehydrostephanine (**4a**) displayed antiarrhythmic effects in the CHCl_3_-induced model. Among them, 7.5 mg/kg of **2b** was able to significantly reduce the incidence of VF induced by CHCl_3_ (*p* < 0.05), increase the number of rats that resumed sinus rhythm from arrhythmia, induced by BaCl_2_ (*p* < 0.01), and the number of rats that maintained sinus rhythm for more than 20 min (*p* < 0.01). Therefore, **2b** showed remarkably higher antiarrhythmic activity and a lower toxicity (LD_50_ = 59.62 mg/kg, mice), simultaneously, indicating that **2b** could be considered as a promising candidate in the treatment of arrhythmia. Structural-activity analysis suggested that variationsin antiarrhythmic efficacy and toxicity of aporphines were related to the C-1,C-2-methylenedioxy group on ring A, restricted ring B structural conformation, *N*-quaternization of ring B, levoduction of 6a in ring C, and the 8-, 9-, 10-methoxy groups on ring D on the skeleton.

## 1. Introduction

Although radio frequency ablation and other new techniques have been applied in clinical therapy for the treatment of cardiac arrhythmia, drug therapy is still an important and essential method of treatment. According to current methodology of the Vaughan Williams’ electrophysiological classification, available anti-arrhythmic drugs are divided into four categories: Na^+^ channel blockers (category I), β-receptor blockers (category II), K^+^ channel blockers (category III), and Ca^+^ channel blockers (category IV) [1]. Unfortunately, many of the drugs that belong to these four categories have also shown unavoidable side-effects, such as cardiac arrhythmia [2]. However, evidence shows that the new category III drugs, working through multiple ion (I_kr_, I_ks_, I_to_, I_Na_) channels, are likely to become the most prospective candidates for the treatment of arrhythmia, due to their higher efficiency, as well as their lower risk of side-effects [3]. Therefore, searching for new components from natural products and their derivatives that act on multiple ion channels seems to be one of the more important approaches in the development of new antiarrhythmic drugs [4].

In the last 30 years, several aprophine-type compounds, such as isocorydine, l-dicentrine, and crebanine, extracted from plants in the subgenus *Tuberiphania* Lo et M. Yang and the genus *Stephania*, *Menispermaceae*, have been found possessing remarkable antiarrhythmic activities [5,6,7]. Based on traditional medical records, some species from this subgenus have been used as medicinal herbs for a long time in Southwest China, with medical functions such as pain-relief, heat-clearing, detoxing, and activating blood circulation [8]. Studies have shown that almost all species of this subgenus contain various types of isoquinoline alkaloids, including aprophines, morphinans, protoberberines, etc., which display extensive physiological activities. They show activity on the K^+^, Ca^2+^, and Na^+^ channel, and some of them have been reported to be blockers of 5-hydroxytryptamine and dopamine receptors [9]. Meanwhile, isoquinoline alkaloids, for example, berberine, *l*-tetrahydropalmatine, and isocorydine, have been used in clinical treatment of cardiovascular and cranial disease in China. The functions on multiple K^+^, Ca^2+^, and Na^+^ ion channels that these alkaloids exhibit coincides with the current leading trend in the screening of new category III anti-arrhythmic drugs [10,11,12]. In our previous observations, crebanine, an aprophine type alkaloid, showed a higher anti-arrhythmic effect on experimental arrhythmia models compared with isocroydine and l-dicentrine [13]; however, it was also found that the acute toxicity of crebanine was higher than that of isocroydine and l-dicentrine. The effective arrhythmia dosage (2.5~5.0 mg/kg) of crebanine was close to its LD_50_ (9.38 mg/kg) in mice [14], which implies that crebanine has a relatively narrow therapeutic window.

The present study is a further investigation regarding the structural modification and evaluation of the arrhythmic activities of aprophine alkaloids. The authors have attempted to find new aprophine derivatives with higher antiarrhythmia efficiencies and lower toxicities. A series of derivatives from three kinds of aporphine (crebanine, isocroydine, and stephanine) were designed and synthesized by connecting specific pharmacophores to enlarge the target distribution of antiarrhythmia. At the same time, hydrophobic groups or lipophilic groups were introduced in order to improve the lipid-water partition coefficient and membrane permeability. The antiarrhythmic effects of the derivatives were preliminarily evaluated in a mouse model of ventricular fibrillation (VF), induced by CHCl_3_, and further confirmed in a rat model of arrhythmia, induced by BaCl_2_. Six derivatives were obtained, and are reported here for the first time, with antiarrhythmic effects and the structure-activity relationships are discussed.

## 2. Results and Discussion

**Scheme 1:**
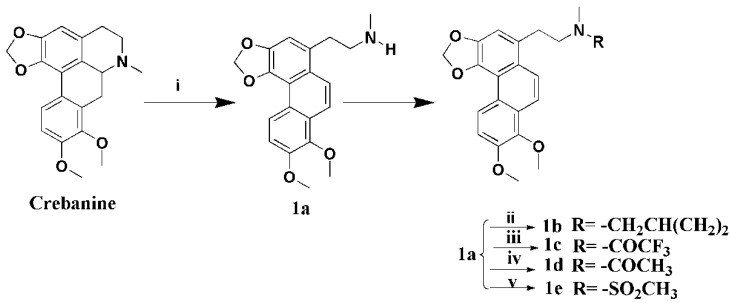
Synthesis of crebanine derivatives **1a**, **1b**, **1c**, **1d**, and **1e**. Reagents and conditions: (**i**) 1-chloroethyl chloroformate, K_2_CO_3_, 1,2-CH_2_ClCH_2_Cl, 85 °C, 6 h, MeOH, 37% HCl, refluxed for 2 h; (**ii**) cyclopropylmethyl bromide, K_2_CO_3_, MeCN, 80 °C, 4 h; (**iii**) trifluoroacetic anhydride, Et_3_N, anhydrous CH_2_Cl_2_, 2 h, r.t.; (**iv**) acetic anhydride, 4-dimethylaminopyridine, Et_3_N, anhydous CH_2_Cl_2_, r.t., 4 h; and (**v**) methanesulfonyl chloride, Et_3_N, anhydrous CH_2_Cl_2_, r.t., 2 h.

**Scheme 2:**
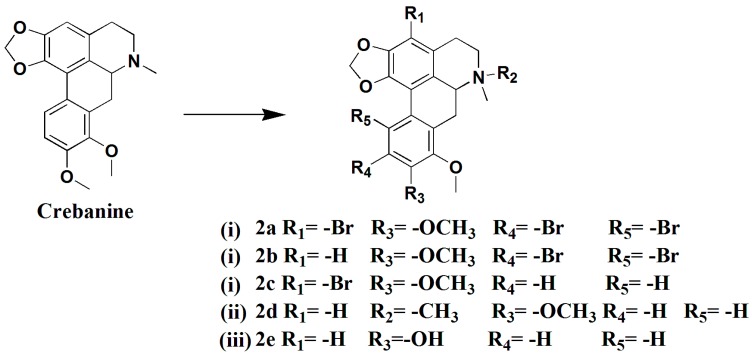
Synthesis of crebanine analogues **2a**, **2b**, **2c**, **2d**, and **2e**. Reagents and conditions: (**i**) *N*-Bromosuccinimide, trifluoroacetic acid, r.t., 18 h; (**ii**) ether, CH_3_I, MeOH, r.t., 16 h; and (**iii**) AlBr_3_, anhydrous CH_2_Cl_2_, nitrobenzene, 5 °C, argon, 18 h.

**Scheme 3:**
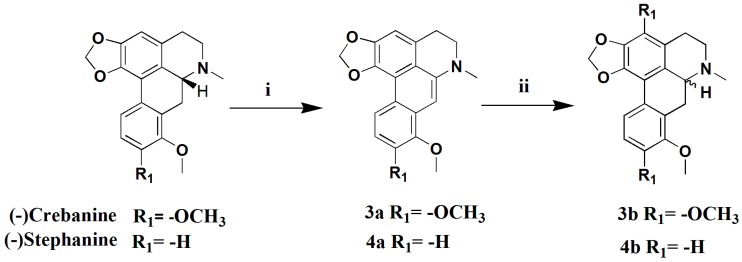
Synthesis of crebanine and stephanine derivatives **3a**, **3b**, **4a** and **4b**. Reagents and conditions: (**i**) 10% Pd/C, MeCN, N_2_, refluxed for 6 h; and (**ii**) NaCNBH_3_, EtOH, 2NHCl, r.t., 18 h.

**Scheme 4:**
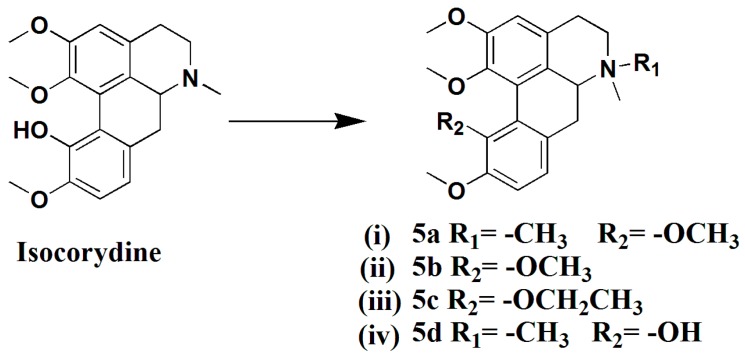
Synthesis of isocorydine derivatives **5a**, **5b**, **5c**, and **5d**. Reagents and conditions: (**i**) Tetrabutylammonium bromide, K_2_CO_3_, anhydrous THF, CH_3_I, r.t., 8 h; (**ii**) N, *N*-diisopropylethylamine, trimethylsilyldiazomethane, MeCN, MeOH, 15 h, r.t.; and (**iii**) Tetrabutylammonium bromide, K_2_CO_3_, anhydrous THF, CH_3_CH_2_I, r.t., 8 h; and (**iv**) ether, CH_3_I, MeOH, r.t., 16 h.

**Scheme 5:**
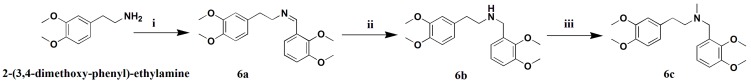
Synthesis of crebanine analogues **6c**. Reagents and conditions: (**i**) 2,3-dimethoxy-benzoic acid , methylbenzene, 120 °C, 8 h; (**ii**) 5%Pd/C, MeOH, H_2_, 1 bar, 8 h; and (**iii**) K_2_CO_3_, anhydrous THF, CHI, r.t., 4 h.

Nineteen products, including 13 derivatives of crebanine, four derivatives of isocorydine, and two derivatives of stephanine, were synthesized, isolated, purified, and identified (Scheme 1, Scheme 2, Scheme 3, Scheme 4 and Scheme 5), of which 11 are reported for the first time: Secocrebanine (**1a**), *N*-cyclopropylmethylsecocrebanine (**1b**), *N*-trifluoroacetamidesecocrebanine (**1c**), *N*-acetamidesecocrebanine (**1d**), *N*-methylsulfonamidesecocrebanine (**1e**), 3,10,11-Tribromocrebanine (**2a**), 10,11-Dibromocrebanine (**2b**), 3-Bromocrebanine (**2c**), 11-Methoxy-*N*-methylisocorydine (**5a**), 11-Methoxyisocorydine (**5b**), and 11-Ethoxyisocorydine (**5c**).

**Figure 1:**
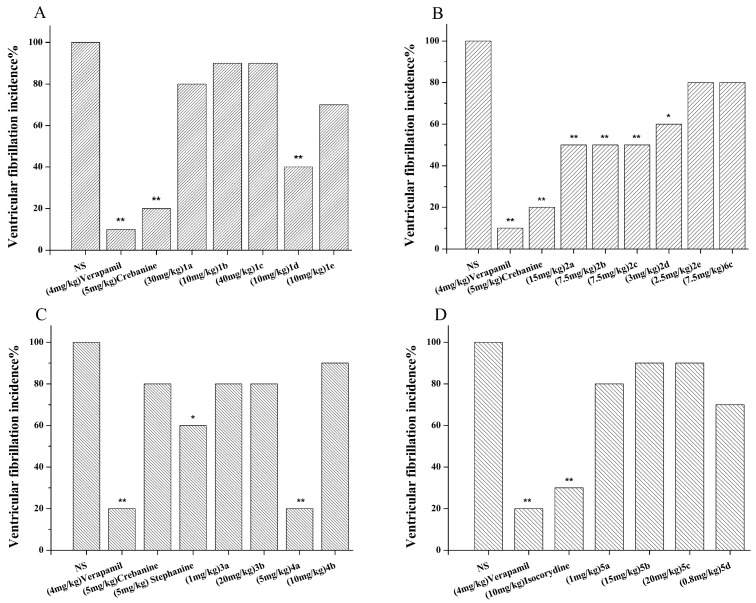
(**A**–**D**) Antiarrthymic activity of 19 aporphine derivatives in a mouse VF model, induced by CHCl_3_ (*n* = 10). Data represent the incidence percentage of VF in 10 experiments. Significantly different from the control group, * *p <* 0.05, ** *p <* 0.01. **1d**, **2a**, **2b**, **2c**, **2d**, and **4a** possess significant antiarrhythmic effects.

The antiarrhythmic activities of the 19 products, respectively, were tested in the mouse model of ventricular fibrillation (VF), induced by CHCl_3_. Six compounds, **1d**, **2a**, **2b**, **2c**, *N*-methylcrebanine (**2d**), and dehydrostephanine (**4a**), were found to possess significant antiarrhythmic effects (*p* < 0.05) (Figure 1A–D). Furthermore, in the rat model of arrhythmia, induced by BaCl_2_, **2a**, **2b**, **2c**, **2d**, and **4a** demonstrated significant antiarrhythmia activity (Table 1). Lastly, in order to evaluate toxicity, the LD_50_ of three active compounds, two bromo-substituted products of crebanine (**2a**, **2b**) and *N*-methylcrebanine (**2d**), were calculated using the sequential method in mice (Table 2) as 59.62, 65.15, and 6.432 mg/kg, respectively.

### 2.1. Opening up of the N-C Bondon Ring B and N-Substitution and Antiarrhythmic Activity/Toxicity

Previously, we characterized some new type III anti-arrhythmic drugs, such as dronedarone [15], most of which contained the *N*-substitution structure [3]. Therefore, we presumed that the *N*-substitution in ring B of aporphine might be one of the key reasons for their anti-arrhythmic activities. Five phenanthrene compounds were synthesized (Scheme 1) by opening up the N-C bond (**1a**) on ring B of crebanine, further connecting the N position with four kinds of lipophilic groups (**1b**–**1e**). The five obtained analogues were tested in the mouse model of VF, induced by CHCl_3_ (Figure 1A). The result showed that, except for compound **1d** (10 mg/kg), which was able to return the occurrence of VF to a normal sinus rhythm in 60% of tested mice (*p* < 0.01) (Figure 1A), the remaining four ring B-opening derivatives (**1a**, **1b**, **1c**, and **1e**) showed no significant effects on inhibiting rapid VF in mice, even though the test dosages (10–40 mg/kg) were up to eight times higher than the dosage of crebanine (5 mg/kg). Simultaneously, the tested mice also showed no obvious toxic symptoms. These results indicate that antiarrhythmic efficiency and toxicity were both decreased when ring B of crebanine was opened up. Therefore, it is speculated that a proper, closed ring B structure of aporphine was necessary, as the flat structure of the restricted ring B structural conformation could embed into myocardial cells more easily than the ring B-opening structure of phenanthrenes.

### 2.2. The Lipophilicity/Hydrophilicity of Crebanine Derivatives and Antiarrhythmic Activity/Toxicity

Bromo-substituted derivatives often increase the lipophilicity of compounds. Bromine atoms were first introduced into the structure of crebanine (Scheme 2) to explore the relationship between lipophilicity and the antiarrhythmic activity/toxicity of aporphines. Compared with normal saline, compounds **2a**, **2b**, and **2c**, three kinds of crebanine bromoderivatives, exhibited a remarkable recovery in mouse VF (*p* < 0.05) (Figure 1B). Notably, dibromide **2b** of crebanine displayed better antiarrhythmic activity than tribromide of crebanine **2a** and monobromide of crebanine **2c** (Table 1). After a 7.5 mg/kg dosage of **2b** was given, the number of animals that recovered, and the number of animals maintaining sinus rhythm ≥20 min was obviously higher than those of **2a** (15 mg/kg) and **2c** (7.5 mg/kg) in the rat model of arrhythmia, induced by CHCl_3_ (Figure 1B). The results indicate that the different numbers of Br replacements varied the lipid solubility of crebanine and changed the level of antiarrhythmic efficiency. Furthermore, the LD_50_ of **2c** (65.15 mg/kg) and **2b** (59.62 mg/kg) in mice (Table 2) were much higher than that of crebanine (LD_50_ 9.38 mg/kg) [14], which showed that toxicity of brominated crebanine was much lower than that of crebanine. This predicts that brominated crebanine may be a prospective modified structure and would serve as a lead compound for antiarrhythmia.

On the other hand, *N*-methylation (quaternization) was introduced into the structure of crebanine in order to determine how the improvement of hydrophilicity in aporphines impacted the antiarrhythmic activity and toxicity. The quaternary ammonium of crebanine (**2d**, Scheme 2) displayed a notable antiarrhythmic effect, even at a lower dosage of 3 mg/kg (Figure 1B), but the tested mice showed symptoms such as polypnea, mydriasis, leaping, and tremors until death, which indicate toxicity of the central nervous system (CNS) and the motor nervous system [16]. The LD_50_ of **2d** was calculated to be 6.432 mg/kg in mice (Table 2), much lower than that of crebanine (9.382 mg/kg), which indicated that *N*-quaternizated aporphines could maintain anti-arrhythmic activity, but that the toxicity was increased at the same time.

### 2.3. Ring D O-Demethylation and Antiarrhythmic Activity

The experiments were designed to remove the *O*-methyl groups in ring D of crebanine in order to acquire more aporphine derivatives (Scheme 2). Table 3 shows all of reactions and their derivatives [17,18,19,20,21,22]. Since ring B was unstable and easy to open using strong acids, it was difficult to cleave the *O*-methyl groups in ring D when only strong acids (48%HBr/CH_3_COOH, 48%HBr, and IH) were used [17,18,19]. Otherwise, when crebanine was treated with BBr_3_ in dichloromethane, a molecular ion peak of the products was observed at *m/z* 300 [M + H]^+^, which implies that both the *O*-methyl groups and methylenedioxy of crebanine had been removed simultaneously. Meanwhile, the target product of the four phenolic hydroxyl groups was not acquired due to its high hydrophilicity and instability. A 9-demethyl group derivative, namely stesakine (**2e**, Scheme 2) [20] in Table 3, was obtained by utilizing the Lewis acids, BCl_3_, and AlBr_3_ [21,22], which indicated that the 9-methoxy group in ring D of crebanine was less stable than the 8-methoxy group.

Compound **2e** showed weak activity at a maximum dissolvable dosage (2.5 mg/kg) in the mouse VF model (Figure 1B), thus, the 9-methoxy group in ring D might be one of the functional groups in aporphine alkaloids. Similarly, the 8- and/or 9- or 10-methoxylation in ring D of the aporphine skeleton, such as crebanine, stephanine, and l-isocrydine, showed notable antiarrhythmic activity. On the contrary, the removal of the 8- or 9- or 10-methyl groups of aporphine alkaloids in ring D may reduce antiarrhythmic activity.

### 2.4. Racemization of Aporphines and Antiarrhythmic Activity

Two racemic compounds of crebanine and stephanne, namely (±) crebanine (**3b**) and (±) stephanine (**4b**), were synthesized by reduction after dehydrogenation of 6a-H (Scheme 3) [23]. Both compounds did not significantly manifest antiarrhythmic activity in the mouse model of VF induced by CHCl_3_ (Figure 1C). The results indicated that the levoduction of the 6a configuration was a functional group for maintaining antiarrhythmic activity.

### 2.5. 6a-H Dehydrogenated Derivatives of Aporphines and Antiarrhythmic Activity/Toxicity

Dehydrocrebanine (**3a**) was derived from dehydrogenating in 6a-H of crebanine and the formation of a double bond in ring C of crebanine (Scheme 3). **3a** showed a very high toxicity in animal tests. In order to avoid animal poisoning and death, the test dosage had to be decreased to 1 mg/kg (Figure 1C); however, antiarrhythmic activity was not obviously observed in the CHCl_3_-induced VF model either. On the other hand, stephanine and its derivative of 6a-H dehydrogenation (dehydrostephanine, **4a**) (5 mg/kg), was able to notably confront VF, caused by CHCl_3_, in mice (Figure 1C). In the rat model of arrhythmia induced by BaCl_2_, both stephanine and **4a** increased the number of animals that resumed normal sinus rhythm (*p <* 0.01), and significantly shortened the time for recovery. However, **4a** was unable to maintain a sinus rhythm, even after more than 3 min post recovery (Table 1), and most of the tested animals died during the observation period (20 min), which implies that the effective duration of **4a** was much shorter and the toxicity was high. These findings may be explained by the fact that dehydrogenation compounds increase the rigidity and conjugacy of the skeleton due to ring C forming a double bond (**3a**, **4a**), which facilitated the infusion of aporphine molecules infusing myocardial cells, and consequently producing much faster and stronger toxic effects relative to the prototype compounds.

### 2.6. 11-Alkylation and Quaternization of Isocorydine

Figure 1D and Table 1 show that isocorydine (10 mg/kg) displayed lower antiarrhythmic activity tendencies compared to crebanine (5 mg/kg) and stephanine (5 mg/kg). The main difference in these three compounds lies on the 1,2-dimethoxy group of ring A, which exists in crebanine and stephanine, but not in isocorydine. This increase in antiarrhythmic activity may be related to the 1,2-dimethoxy group structure, which widens the planar configuration of molecules.

All of the modified products of isocorydine, **5a**, **5b**, **5c**, and **5d** (Scheme 4) [24,25] were inactive in the model of VF induced by CHCl_3_ (Figure 1D) at appropriate test dosages. Meanwhile, **5a** and **5d** were both *N*-methylated as quaternary ammonium compounds, and presented such a high toxicity that the test dosages had to be reduced to 1 mg/kg (**5a**) and 0.8 mg/kg (**5d**) (Table 1) to avoid animal death. The reactions of toxicity appeared, much like those of *N*-methylcrebanine (**2d**), in the central nervous system and the motor nervous system. The experiments also evidenced that the substitution of the 11-methoxy group (**5b**) or 11-ethoxy group (**5c**) of isocorydine did not have any benefits for antiarrhythmic activity.

### 2.7. Design of An Analogue of Crebanine

An analogue of crebanine, compound **6c**, was designed and synthesized according to the key group of crebanine (Scheme 5), which contained two basic sites, a tertiary amine group and a dimethoxy-benzene group. However, animal tests showed that **6c** could not significantly inhibit the occurrence of VF induced in mice. Therefore, it is necessary to design a series of dimethoxy-phenylamine derivatives in order to seek out the potential active compound.

## 3. Experimental Section

### 3.1. Synthesis

#### 3.1.1. General Information

Isocorydine, crebanine, and stephanine were extracted from the plant, identified as *Stephania* yunnanensis Lo by Professor Yunshu Ma, Yunnan University of Traditional Chinese Medicine, and as reported previously [26,27]. Solvents and reagents used in syntheses were of analytical grade, were purchased from commercial sources, and used without further purification.

All reactions were carried out under an atmosphere of argon, with magnetic stirring, in flame dried or oven-dried glassware. Analytical thin layer chromatography was performed on 0.25-mm silica gel GF 254 plates (Qingdao Haiyang Chemical Co., Ltd., Qingdao, China). Visualization was accomplished with UV light and bismuth potassium iodide solution staining followed by heating. 1H-NMR spectra were recorded on a 400 MHz spectrometer (Amersham Pharmacia Biotech AB Inc., Tokyo, Japan) in CDCl_3_ or CD_3_OD at ambient temperature, using the solvent signal as an internal standard. Data are reported as: (br/broad, s/singlet, d/doublet, t/triplet, q/quartet, m/multiplet; integration; coupling constant(s) in hertz). ^13^C-NMR spectra were recorded on a 400 MHz spectrometer (Amersham Pharmacia Biotech AB Inc., Tokyo, Japan) in CDCl_3_ at ambient temperature, using the solvent signal as an internal standard. Electrospray ionization mass spectrometry was detected with a Brukerama Zon SL (Bruker Daltonics Inc., Leipzig, Bremen, Germany); HR-MS was detected with 6200 series TOF/6500 series (Agilent Technologies Inc., Santa Clara, UT, USA); rotation was detected with a WZZ-three digital automatic polarimeter (Shanghai Shengguang Technology Co., Ltd., Shanghai, China).

#### 3.1.2. Preparation of Secocrebanine (**1a**)

A mixture of crebanine (2 g, 5.9 mmol) and K_2_CO_3_ (1.7 g, 1.2 mmol) in 1,2-dichloroethane (20 mL) was stirred and 1-chloroethyl chloroformate (1.0 g, 7.1 mmol) was added slowly. The mixture was heated to 85 °C for 6 h, the 1,2-dichloroethane-insoluble material was filtered off, and the filtrate was concentrated to give the residue, which was dissolved in MeOH (20 mL) and five drops of 37% HCl, and refluxed for 2 h. The solution was concentrated and neutralized with saturated Na_2_CO_3_, and the residue was extracted with CH_2_Cl_2_. The combined extracts were dried over anhydrous Na_2_SO_4_, filtered, and concentrated to get a crude product, which was crystallized from EtOAC at 4 °C, yielding compound **1a** (1.2 g, 60%) as a pink-white solid. ^1^H-NMR (400 MH_Z_, CDCl_3_, δ; ppm) 8.83 (d, *J =* 9.2 H_Z_, 1H, 5-H), 7.93 (d, *J =* 9.6 H_Z_, 1H, 10-H), 7.87 (d, *J =* 9.6 H_Z_, 1H, 9-H), 7.29 (d, *J =* 8.8 H_Z_, 1H, 6-H), 7.10 (s, 1H, 2-H), 6.22 (s, 2H, -OCH_2_O-), 4.02 (s, 3H, 8-OCH_3_), 4.00 (s, 3H, 9-OCH_3_), 3.28–3.24 (t, *J =* 7.2 H_Z_, 2H, -CH_2α_), 2.97–2.93 (t, *J =* 7.2 H_Z_, 2H, -CH_2β_), 2.47 (s, 3H, -NCH_3_); ^13^C-NMR (100 MH_Z_, CDCl_3_, δ; ppm) 33.87 (t, -CH_2β_), 36.43 (q, N-CH_3_), 53.12 (t, -CH_2α_), 56.30 (q, 7-OCH_3_), 61.32 (q, 8-OCH_3_), 100.95 (t, -OCH_2_O-), 110.05 (d, C-2), 112.60 (d, C-6), 117.11 (s, C-8a), 118.38 (d, C-5), 123.26 (s, C-4a), 123.64 (d, C-9), 123.79 (d, C-10), 125.19 (s, C-1a), 127.41 (s, C-5a), 130.88 (s, C-1), 141.76 (s, C-4), 143.23 (s, C-7), 144,98 (s, C-3), 149.96 (s, C-8), Positive ESI-MS *m*/*z*: 340.2 [M + H]^+^. HR-MS for C_20_H_21_NO_4_ [M + H]^+^; calcd. 340.1543, found: 340.1542.

#### 3.1.3. Preparation of *N*-Cyclopropylmethylsecocrebanine (**1b**)

Product **1a** (200 mg, 0.59 mmol), K_2_CO_3_ (163 mg, 1.18 mmol), and cyclopropylmethyl bromide (96 mg, 0.71 mmol) were added to MeCN (20 mL) and heated at 80 °C for 4 h. The reaction mixture was filtered and concentrated to give a residue, which was purified by silica column chromatography (CHCl_3_/MeOH 80:1), yielding compound **1b** (180 mg, 78%) as a white solid. ^1^H-NMR (400 MH_Z_, CDCl_3_, δ; ppm) 8.81(d, *J =* 9.2 H_Z_, 1H, 5-H), 8.04 (d, *J =* 9.6 H_Z_, 1H, 10-H), 7.93 (d, *J =* 9.2 H_Z_, 1H, 9-H), 7.31 (d, *J =* 8.4 H_Z_, 1H, 6-H), 7.16 (s, 1H, 2-H), 6.23 (s, 2H, -OCH_2_O-), 4.02 (s, 3H, 8-OCH_3_), 3.99 (s, 3H, 9-OCH_3_), 3.84–3.78 (m, 1H, CH_2α_), 3.66–3.60 (m, 1H, -CH_2β_), 3.47–3.42 (m, 1H, -CH_2β_), 3.18–3.15 (m, 2H, N-CH_2_), 2.97 (s, 3H, -NCH_3_), 2.93–2.89 (m, 1H, CH_2α_), 1.30–1.25 (m, 1H, -CH), 0.85–0.82 (m, 2H, -CH_2_), 0.50–0.47 (m, 1H, -CH_2_). .^13^C-NMR (100 MH_Z_, CDCl_3_, δ; ppm) 100.62 (t, -OCH_2_O-), 52.82 (t, -CH_2β_), 29.59 (t, CH_2α_), 29.26 (t, -CH_2_), 29.05 (t, -CH_2_), 61.00 (q, 8-OCH_3_), 56.02 (q, 8-OCH_3_), 43.97 (q, N-CH_3_), 115.77 (d, C-5), 114.88 (d, C-6),62.40(d, -CH), 152.55 (s, C-8), 145.17 (s, C-3), 145.02 (s, C-7), 141.99 (s, C-4), 133.19 (s, C-1),130.69 (s, C-5a), 125.73(s, C-1a), 124.66 (s, C-4a), 114.88(s, C-8a). Positive ESI-MS *m*/*z*: 394.2 [M + H]^+^. HR-MS for C_24_H_27_NO_4_ [M + H]^+^; calcd. 394.2013, found: 394.2026.

#### 3.1.4. Preparation of *N*-Trifluoroacetamidesecocrebanine (**1c**)

A solution of **1a** (200 mg, 0.59 mmol) and Et_3_N (72 mg, 0.71 mmol) in anhydrous CH_2_Cl_2_ (30 mL), trifluoroacetic anhydride (149 mg, 0.71 mmol) was slowly added under an ice-bath, then, the solution was stirred for 2 h at room temperature. The mixture was diluted with water (20 mL) and extracted with CH_2_Cl_2_ (3 × 10 mL). The combined extracts were dried over anhydrous Na_2_SO_4_, filtered and concentrated to give a crude product, which was purified by silica gel column chromatography (CH_2_Cl_2_/MeOH 100:1), and yielding compound **1c** (210 mg, 82%) as a white solid. ^1^H-NMR (400 MH_Z_, CDCl_3_, δ; ppm) 8.83 (d, *J =* 9.2 H_Z_, 1H, 5-H), 7.99 (d, *J =* 9.2 H_Z_, 1H, 10-H), 7.94 (d, *J =* 9.6 H_Z_, 1H, 9-H), 7.31 (d, *J =* 9.2 H_Z_, 1H, 6-H), 7.07 (s, 1H, 2-H), 6.24 (s, 1H, -OCH_2_O-), 6.23 (s, 1H, -OCH_2_O-), 4.03 (s, 3H, 8-OCH_3_), 4.00 (s, 3H, 9-OCH_3_), 3.73–3.69 (t, *J =* 7.4 H_Z_, 2H, -CH_2α_), 3.36–3.32 (t, *J =* 7.2 H_Z_, 2H, -CH_2β_), 3.11 (s, 1H, -NCH_3_), 2.99 (s, 2H, -NCH_3_). ^13^C-NMR (100 MH_Z_, CDCl_3_, δ; ppm) 30.67 (t, CH_2α_), 36.04 (q, N-CH_3_), 51.79 (t, -CH_2β_ ), 56.32 (q, 7-OCH_3_), 61.33(q, 8-OCH_3_), 101.21 (t, -OCH_2_O-), 110.13 (d, C-2), 112.91 (d, C-6), 117.15 (s, C-8a), 119.12 (s, C-4a), 119.28 (d, C-5), 122.08 (s, CF_3_), 123.77 (d, C-9), 123.81 (d, C-10), 125.39 (C-1a), 127.47 (C-5a), 128.83 (s, C-1), 142.27 (s, C-4), 143.32 (s, C-7), 145.08 (s, C-3), 150.15 (s, C-8). Positive ESI-MS *m*/*z*: 458.2 [M + Na]^+^. HR-MS for C_22_H_20_F_3_NO_5_ [M + Na]^+^; calcd. 458.1186, found: 458.1188.

#### 3.1.5. Preparation of *N*-Acetamidesecocrebanine (**1d**)

To a stirred solution of **1a** (200 mg, 0.59 mmol), 4-dimethylaminopyridine (144 mg, 1.18 mmol), and Et_3_N (72 mg, 0.71 mmol) in anhydrous CH_2_Cl_2_ (20 mL), acetic anhydride (72 mg, 0.71mmol) was slowly added and stirred for 4 h at room temperature. The mixture was diluted with water (10 mL) and extracted with CH_2_Cl_2_ (3 × 10mL). The combined extracts were dried over anhydrous Na_2_SO_4_, filtered, and concentrated to get a crude product, which was purified by silica gel column chromatography (CH_2_Cl_2_/MeOH 100:1), yielding compound **1d** (180 mg, 80%) as a white solid. ^1^H-NMR (400 MH_Z_, CDCl_3_, δ; ppm) 8.83 (d, *J =* 8.4 H_Z_, 1H, 5-H), 8.00 (d, *J =* 10.0 H_Z_, 1H, 10-H) 7.96 (d, *J =* 9.6 H_Z_, 1H, 9-H), 7.32 (d, *J =* 9.2 H_Z_, 1H, 6-H), 7.09 (s, 1H, 2-H), 6.23 (s, 1H, -OCH_2_O-), 6.22 (s, 1H, -OCH_2_O-), 4.03 (s, 6H, 8-OCH_3_), 4.00 (s, 6H, 9-OCH_3_), 3.67–3.61 (dd, *J =* 15.6 H_Z_, *J =* 8.0 H_Z_, 2H, -CH_2α_), 3.31–3.27 (t, *J =* 7.4 H_Z_, 2H, -CH_2β_), 3.00 (s, 1H, -NCH_3_), 2.81 (s, 2H, -NCH_3_), 2.09 (s, 2H, -NOCH_3_), 1.85 (s, 1H, -NOCH_3_). ^13^C-NMR (100 MH_Z_, CDCl_3_, δ; ppm) 22.03 (q, -NOCH_3_), 32.57 (t, -CH_2α_), 37.39 (q, N-CH_3_), 51.99 (t, -CH_2β_), 56.33 (q, 7-OCH_3_), 61.32 (q, 8-OCH_3_), 100.08 (t, -OCH_2_O-), 110.28 (d, C-2), 112.88 (d, C-6), 117.17 (s, C-8a), 119.10 (d, C-5),123.76 (d, C-9), 123.81 (d, C-10), 125.55 (s, C-4a), 127.50 (C-1a), 128.72 (C-5a), 130.31 (s, C-1), 142.30 (s, C-4), 143.28 (s, C-7), 145.14 (s, C-3), 150.11 (s, C-8), 170.69 (s, -C=O).Positive ESI-MS *m*/*z*: 404.2 [M + Na]^+^.HR-MS for C_22_H_23_NO_5_ [M + Na]^+^; calcd. 404.1468, found: 404.1474.

#### 3.1.6. Preparation of *N*-Methylsulfonamidesecocrebanine (**1e**)

To a mixture of **1a** (200 mg, 0.59 mmol) and Et_3_N (72 mg, 0.71 mmol) in anhydrous CH_2_Cl_2_ (30 mL) under an ice-bath, methanesulfonyl chloride (81 mg, 0.71 mmol) was slowly added and stirred for 2 h at room temperature. The mixture was diluted with water (20 mL) and extracted with CH_2_Cl_2_ (3 × 10 mL). The combined extracts were dried over anhydrous Na_2_SO_4_, filtered, and concentrated to give a crude product, which was purified by silica gel column chromatography (CH_2_Cl_2_/MeOH 100:1), yielding **1e** (210 mg, 82%) as a white solid. ^1^H-NMR (400 MH_Z_, CDCl_3_, δ; ppm) 8.83 (d, *J =* 9.2 H_Z_, 1H, 5-H), 7.98 (d, *J =* 9.6 H_Z_, 1H, 10-H), 7.85 (d, *J =* 9.6 H_Z_, 1H, 9-H), 7.31(d, *J =* 9.2 H_Z_, 1H, 6-H), 7.11 (s, 1H, 2-H), 6.23 (s, 2H, -OCH_2_O-), 4.03 (s, 3H, 8-OCH_3_), 4.00 (s, 3H, 9-OCH_3_), 3.46–3.42 (m, 2H, -CH_2α_), 3.37–3.34 (m, 2H, -CH_2β_), 2.92 (s, 3H, -NCH_3_), 2.74 (s, 3H, -NSO_2_CH_3_). ^13^C-NMR (100 MH_Z_, CDCl_3_, δ; ppm) 33.26 (t, -CH_2α_), 35.38 (t, -NSO_2_CH_3_), 51.56 (t, -CH_2β_), 56.31 (q, 7-OCH_3_), 61.34 (q, 8-OCH_3_), 101.10 (t, -OCH_2_O-), 110.30 (d, C-2), 112.76 (d, C-6),117.13 (s, C-8a), 119.06 (d, C-5),122.72 (d, C-9),123.77 (d, C-10),125.21 (s, C-4a),127.40 (C-1a), 128.88 (s, C-1), 142.20 (s, C-4),143.29 (s, C-7), 145.07 (s, C-3),150.07 (s, C-8). Positive ESI-MS *m*/*z*: 440.2 [M + Na]^+^. HR-MS for C_21_H_23_NO_6_S [M + Na]^+^; calcd. 440.1138, found: 440.1140.

#### 3.1.7. Preparation of 3,10,11-Tribromocrebanine (**2a**), 10,11-Dibromocrebanine (**2b**) and 3-Bromocrebanine (**2c**)

Crebanine (200 mg, 0.59 mmol) and *N*-Bromosuccinimide (213.6 mg, 1.2 mmol) were added to trifluoroacetic acid (5 mL), the mixture was stirred for 18hat room temperature, diluted with water (10 mL), and neutralized with saturated NaHCO_3_ and extracted with CH_2_Cl_2_ (3 × 10 mL). The combined CH_2_Cl_2_ solution was brined with water, dried over anhydrous Na_2_SO_4_, filtered, and concentrated to give a crude product, which was purified by silica gel column chromatography (CH_2_Cl_2_/MeOH 200:1→100:1), yielding compound **2a** (52 mg, 27%), compound **2b** (50 mg, 17%), and compound **2c** (80 mg, 33%).

Compound **2a,** pale yellow solid. ^1^H-NMR (400 MH_Z_, CDCl_3_, δ; ppm) 6.16 (1H, s, 9, -OCH_2_O-), 6.06 (1H, s, -OCH_2_O-), 3.93 (3H, s, 8-OCH_3_), 3.87 (3H, s, 9-OCH_3_), 3.54 (1H, d, *J =* 12.8 H_Z_, 6a-H), 3.16–3.09 (m, 1H, 7-Ha), 2.99–2.91 (m, 2H, 5-Ha, 7-Hb), 2.77–2.72 (m, 1H, 5-Ha), 2.60 (s, 3H, -NCH_3_), 2.53–2.50 (m, 1H, 4-Ha), 2.15–2.02 (m, 1H, 4-Hb). ^13^C-NMR (100 MH_Z_, CDCl_3_, δ; ppm), 29.29 (t, C-7), 29.33 (t, C-4), 52.78 (t, C-5), 44.02 (t, -NCH_3_), 60.82 (q, 9-OCH_3_), 61.17 (q, 8-OCH_3_), 62.18 (d, C-6a), 100.77 (t, -OCH_2_O-),102.96 (s, C-3), 115.29 (s, C-10), 118.63 (s, C-11), 125.78 (s, C-7a), 129.83 (s, C-1b), 130.73 (s, C-3a), 132.67 (s, C-11a), 142.26 (s, C-1), 145.28 (s, C-8), 149.72 (s, C-2), 150.88 (s, C-9a). Positive ESI-MS *m*/*z*: 579.2 [M + 3]^+^. HR-MS for C_20_H_18_Br_3_NO_4_ [M + H]^+^; calcd.573.8859, found:573.8853.

Compound **2b,** white solid. ^1^H-NMR (400 MH_Z_, CDCl_3_, δ; ppm) 7.13 (s, 1H, 3-H), 6.14 (s, 1H, -OCH_2_O-), 6.05 (s, 1H, -OCH_2_O-), 3.89 (s, 3H, 8-OCH_3_), 3.79 (s, 3H, 9-OCH_3_), 3.61–3.57 (m, 1H, 6a-H), 3.11–3.07 (m, 1H, 7-Ha), 2.97–2.87 (m, 2H, 5-Ha, 7-Hb), 2.80–2.66 (m, 1H, 5-Hb), 2.56 (s, 3H, -NCH_3_), 2.52–2.47 (m, 1H, 4-Ha), 2.11–2.04 (t, *J =* 13.6 H_Z_, 1H, 4-Hb). ^13^C-NMR (100 MH_Z_, CDCl_3_, δ; ppm) 100.62 (t, -OCH_2_O-), 52.82 (t, C-5), 29.26 (t, C-4), 29.05 (t, C-7), 62.40 (d, C-6a), 115.95 (d, C-3), 152,55 (s, C-9), 145.17 (s, C-2), 145.09 (s, C-8), 141.99 (s, C-1), 133.19 (s, C-11a), 130.69 (s, C-3a), 125.73 (s, C-7a), 124.66 (s, C-1a). Positive ESI-MS *m*/*z*: 498.0 [M + H]^+^. HR-MS for C_20_H_19_Br_2_NO_4_ [M + H]^+^; calcd. 495.9754, found: 495.9759.

Compound **2c,** white solid. Positive ESI-MS *m*/*z*: 418 [M]^+^, 420.0 [M + 2]^+^. ^1^H-NMR (400 MH_Z_, CDCl_3_, δ; ppm) 7.76 (d, *J =* 8.8 H_Z_, 1H, 11-H), 6.90 (d, *J =* 8.8 H_Z_, 1H, 10-H), 6.22 (s, 1H, -OCH_2_O-), 6.07(s, 1H, -OCH_2_O-), 3.91 (s, 3H, 8-OCH_3_), 3.84 (s, 3H, 9-OCH_3_), 3.82–3.80 (m, 1H, 6a-H), 3.09-3.04 (m, 2H, 5-Ha, 7-Hb), 2.90–2.82 (m, 2H, 5-Ha, 7-Hb), 2.17 (s, 3H, -NCH_3_), 1.95–1.87 (m, 1H, 4-Ha), 1.79–1.72 (m, 1H, 4-Hb).

#### 3.1.8. Preparation of *N*-Methylcrebanine (**2d**)

A mixture of crebanine (200 mg, 0.59 mmol), ether (109 mg, 1.48 mmol) and CH_3_I (184.5 mg, 1.3 mmol) in MeOH (10 mL) was stirred for 16 h at room temperature. The reaction mixture was concentrated and extracted with CHCl_3_ (3 × 10 mL). The combined extracts were dried over anhydrous Na_2_SO_4_, filtered, and concentrated to give a crude product, which was purified by silica gel column chromatography (CH_2_Cl_2_/MeOH 30:1), yielding compound **2d** (180 mg, 86%) as a white solid. ^1^H-NMR (400 MH_Z_, CDCl_3_, δ; ppm) 7.70 (d, *J =* 8.8 Hz, 1H, 11-H), 7.05 (d, *J =* 8.8 Hz, 1H, 10-H), 6.78 (s, 1H, 3-H), 6.18 (s, 1H, -OCH_2_O-), 5.98 (s, 1H, -OCH_2_O-), 4.74–4.70 (m, 1H, 6a-H), 3.80 (s, 3H, 8-OCH_3_), 3.76–3.73 (m, 1H, 7-Ha), 3.72 (s, 3H, 9-OCH_3_), 3.64–3.55 (m, 2H, 7-Hb,5-Ha), 3.33 (s, 3H, -NCH_3_), 3.18–3.09 (m, 1H, 5-Hb), 2.97 (s, 3H, -NCH_3_), 2.94–2.89 (m, 1H, 4-Ha), 2.70–2.63 (t, *J =* 14.2 Hz, 1H, 4Hb). Positive ESI-MS *m*/*z*: 354.2 [M]^+^.

#### 3.1.9. Preparation of Stesakine (**2e**)

Crebanine (200 mg, 0.59 mmol) in 2mL of anhydrous CH_2_Cl_2_ was slowly added into a suspension of AlBr_3_ (320 mg, 1.2 mmol) in nitrobenzene (5 mL) at 5 °C under argon. The solution was stirred for 18 h, diluted with water (20 mL) and neutralized with saturated NaHCO_3_. The mixture was extracted with CH_2_Cl_2_ (3 × 10 mL), and the combined CH_2_Cl_2_ solution was brined with water, dried with anhydrous Na_2_SO_4_, filtered, and concentrated to give a crude product, which was purified by silica gel column chromatography (CH_2_Cl_2_/MeOH 50:1), yieldingcompound **2e** (56 mg, 29%) as a colorless oil. ^1^H-NMR (400 MH_Z_, CDCl_3_, δ; ppm) 7.63 (d, *J =* 8.8 H_Z_, 1H, 11-H), 6.83 (d, *J =* 8.8 H_Z_, 1H, 10-H), 6.53 (s, 1H, 3-H), 6.06 (s, 1H, -OCH_2_O-), 5.92 (s, 1H, -OCH_2_O-), 5.77 (s, 1H, 9-OH), 3.93 (s, 3H, 8-CH_3_), 3.69–3.64 (dd, *J =* 16.0 H_Z_, *J =* 4.0 H_Z_, 1H, 6a-H), 3.19–3.03 (m, 3H, 7-H, 5-Ha), 2.65–2.61 (m, 1H, 5-Hb), 2.60 (s, 3H, -NCH_3_), 2.56–2.50 (m, 1H, 4-Ha), 2.32–2.25 (t, *J =* 10.4 H_Z_, 1H, 4-Hb), ^13^C-NMR (100 MH_Z_, CDCl_3_, δ; ppm) 142.2 (s, C-1), 116.7 (s, C-1a), 126.5 (s, C-1b), 146.6 (s, C-2), 106.7 (d, C-3), 126.7 (s, C-3a), 26.3 (t, C-4), 56 (t, C-5), 61.9 (d, 6a), 29.2 (t, C-7), 124.8 (s, C-7a), 156.4 (s, C-8), 145.8 (s, C-9), 108.5 (d, t-10), 118.7 (d, C-11), 126.6 (s, C-11a), 100.1 (t, -CH_2_O-), 44.0 (q, -NCH_3_), 56.0 (q, 8-OCH_3_). Positive ESI-MS *m*/*z*: 326 [M + H]^+^.

#### 3.1.10. Preparation of Dehydrocrebanine (**3a**)

Pd/C (10%, 210 mg) was added to a suspension of crebanine (200 mg, 0.59 mmol) in MeCN (20 mL), and the solution was refluxed for 6 h under N_2_. The mixture reaction solution was filtered and the filtrate was evaporated to give compound **3a** (181 mg, 91%) as a yellowish-green solid. ^1^H-NMR (400 MH_Z_, CDCl_3_, δ; ppm) 8.66 (d, *J* = 8.8 Hz, 1H, 11-H), 7.03 (d, *J* = 9.2 Hz, 1H, 10-H), 6.89 (s, 2H, 3-H, 7-H), 6.19 (s, 2H, -OCH_2_O-), 3.99 (s, 3H, 8-OCH_3_), 3.97 (s, 3H, 9-OCH_3_), 3.34–3.37 (t, *J* = 5.8 Hz, 2H, 5-H), 3.24–3.31 (t, *J =* 5.8 Hz, 2H, 4-H), 3.13 (s, 3H, -NCH_3_). Positive ESI-MS *m*/*z*: 348.2 [M + H]^+^.

#### 3.1.11. Preparation of (±) Crebanine (**3b**)

A mixture of **3a** (150 mg, 0.44 mmol) and NaCNBH_3_ (36 mg, 0.57 mmol) in absolute EtOH was stirred, and a mixture of EtOH and 2NHCl was added until the pH approached 3.0 (within 4 h). Then, the solution was continuously stirred for 18 h at room temperature. After evaporation of the reaction mixture, the pH was adjusted to 8 with saturated Na_2_CO_3_ and the mixture solution was extracted with CH_2_Cl_2_ (3 × 10 mL). The combined extracts were dried over anhydrous Na_2_SO_4_, filtered, and concentrated to get a crude product, which was purified by silica gel column chromatography (CH_2_Cl_2_/MeOH 50:1), yielding compound **3b** (120 mg, 80%) as a yellow oil. ${\left[\alpha \right]}_{D}^{25}$: 0° (C 0.2, MeOH). ^1^H-NMR (400 MH_Z_, CDCl_3_, δ; ppm) 7.81 (d, *J =* 8.8 Hz, 1H, 11-H), 6.88 (d, *J =* 8.8 Hz, 1H, 10-H), 6.53 (s, 1H, 3-H), 6.07 (s, 1H, -OCH_3_O-), 5.90 (s, 1H, -OCH_3_O-), 3.90 (s, 3H, 8-OCH_3_), 3.81 (s, 3H, 9-OCH_3_), 3.70–3.65 (dd, *J =* 14.8 Hz, *J =* 4.4 Hz, 1H, 6a-H), 3.18–3.04 (m, 3H, 7-H, 5-Ha), 2.66–2.61 (m, 1H, 5-Hb), 2.59 (s, 3H, -NCH_3_), 2.56–2.49 (m, 1H, 4-Ha), 2.33–2.26 (t, *J =* 14.2 Hz, 1H, 4-Hb). Positive ESI-MS *m*/*z*: 340.2 [M + H]^+^.

#### 3.1.12. Preparation of Dehydrostephanine (**4a**)

In a similar manner used in the preparation of **3a**, stephanine (200 mg, 0.65 mmol) was subjected to a dehydrogenation reaction to give compound **4a** (162 mg, 81%) as a yellowish-green solid. 1H-NMR (400 MH_Z_, CDCl_3_, δ; ppm) 8.56 (d, *J =* 8.4 H_Z_, 1H, 11-H), 8.29 (d, *J =* 8.0 H_Z_, 10-H), 7.08 (s, 1H, 9-H), 6.96 (s, H, 3-H), 6.93 (s, H, 7-H), 6.21 (s, 2H, -OCH_2_O-), 4.02 (s, 3H, 8-OCH_3_), 3.40–3.37 (t, *J =* 5.6 HZ, 2H, 5-CH_2_), 3.27–3.24 (m, 2H, 5-CH_2_), 3.12 (s, 3H). Positive ESI-MS *m*/*z*: 308.2 [M + H]^+^.

#### 3.1.13. Preparation of (±) Stephanine (**4b**)

In a similar manner used for the preparation of **3b**, **4a** (150 mg, 0.49 mmol) was subjected to a racemization reaction to give compound **4b** (125 mg, 83%) as a yellowish-green solid. ${\left[\alpha \right]}_{D}^{25}$: 0° (C 0.2, MeOH). ^1^H-NMR (400 MH_Z_, CDCl_3_, δ; ppm) 7.70 (d, *J =* 8.0 Hz, 1H, 11-H), 7.31–7.26 (t, *J =* 8.0 Hz, 1H, 10-H), 6.85 (d, *J =* 8.0 Hz, 1H, 9-H), 6.58 (s, 1H, 3-H), 6.10 (s, 1H, -OCH_2_O-), 5.95 (s, 1H, -OCH_2_O-), 3.86 (s, 1H, 8-OCH_3_), 3.78–3.74 (dd, *J =* 14.8 Hz, *J =* 3.6 Hz, 1H, 6a-H), 3.40–3.34 (m, 3H, 7-H, 5a-H), 2.90–2.83 (m, 1H, 5a-H), 2.80 (s, 3H, -NCH_3_), 2.77–2.72 (m, 1H, 4a-H), 2.58–2.51 (t, *J =* 14.4 Hz, 1H, 4b-H). Positive ESI-MS *m*/*z*: 310.2 [M + H]^+^.

#### 3.1.14. Preparation of 11-Methoxy-*N*-Methylisocorydine (**5a**)

To a mixture of isocorydine (200 mg, 0.59 mmol), Tetrabutylammonium bromide (16 mg, 0.03 mmol), and K_2_CO_3_ (163 mg, 1.18 mmol) in anhydrous THF (10 mL) CH_3_I (185 mg, 1.30 mmol) was successively added over a 10-min period and stirred for 8 h at room temperature. The reaction mixture was condensed and extracted with CHCl_3_. The combined extracts were dried over anhydrous Na_2_SO_4_, filtered, and concentrated to give a crude product, which was purified by silica gel column chromatography (CH_2_Cl_2_/MeOH 50:1), yielding compound **5a** (154 mg, 71%) as a white solid. ^1^H-NMR (400 MH_Z_, CDCl_3_, δ; ppm) 6.95 (d, *J =* 8.0 H_Z_, 1H, 8-H), 6.84 (d, *J =* 8.4 H_Z_, 1H, 9-H), 6.66 (s, 1H, 3-H), 3.88 (d, *J =* 4.0 H_Z,_ 6H, 1-OCH_3_,2-OCH_3_), 3.87 (s, 3H,11-OCH_3_), 3.72 (s, 3H, 10-OCH_3_), 3.17–3.10 (m, 1H, 6a-H), 3.04–2.99 (m, 2H, 7-Ha, 5-Ha), 2.87 (d, *J =* 12.4 H_Z_, 1H, 7-Hb), 2.69 (d, *J =* 16.8 H_Z_, 1H, 5-Hb), 2.53–2.48 (m, 4H, -NCH_3_, 4-Ha), 2.40–2.33 (t, *J =* 13.0 H_Z_, 1H, 4-Hb).^13^C-NMR (100 MH_Z_, CDCl_3_, δ; ppm) 29.73 (t, C-4), 23.03 (t, C-7), 42.94 (q, N-CH_3_), 53.07 (q, N-CH_3_), 55.83 (q, 10-OCH_3_), 55.97(q, 2-OCH_3_), 60.00 (t, C-5), 60.21 (q, 1-OCH_3_), 60.40 (q, 11-OCH_3_), 111.46 (d, C-3), 112.72 (d, C-9), 121.63 (s, C-11a). 122.36 (d, C-8), 123.98 (s, C-1a), 123.96 (s, C-7a), 126.18 (s, C-1b), 146.35 (s, C-1), 147.04 (s, C-10), 152.20 (s, C-11), 152.54 (s, C-20). Positive ESI-MS *m*/*z*: 370.2 [M + H]^+^. HR-MS for C_22_H_28_NO_4_ [M + H]^+^; calcd. 370.2013, found: 370.2026.

#### 3.1.15. Preparation of 11-Methoxyisocorydine (**5b**)

A solution of isocorydine (200 mg, 0.59 mmol) in MeCN (5 mL) and MeOH (5 mL) was treated with *N*,*N*-diisopropylethylamine (91 mg, 0.71 mmol) and trimethylsilyldiazomethane (0.36 mL, 2M in hexane), the mixture was stirred at room temperature for 15 h, condensed, and extracted with CHCl_3_. The combined extracts were dried over anhydrous Na_2_SO_4_, filtered, and concentrated to give a crude product, which was purified by silica gel column chromatography (CHCl_3_–MeOH = 80:1), yielding compound **5b** (51 mg, 24%) as a colorless oil. ^1^H-NMR (400 MH_Z_, CDCl_3_, δ; ppm) 6.95 (d, *J =* 8.0 H_Z_, 1H, 8-H), 6.84 (d, *J =* 8.4 H_Z_, 1H, 9-H), 6.66 (s, 1H, 3-H), 3.88 (d, *J =* 4.0 H_Z,_ 6H, 1-OCH_3_,2-OCH_3_), 3.87 (s, 3H,11-OCH_3_), 3.72 (s, 3H, 10-OCH_3_), 3.17–3.10 (m, 1H, 6a-H), 3.04–2.99 (m, 2H, 7-Ha, 5-Ha), 2.87 (d, *J =* 12.4 H_Z_, 1H, 7-Hb), 2.69 (d, *J =* 16.8 H_Z_, 1H, 5-Hb), 2.53–2.48 (m, 4H, -NCH_3_, 4-Ha), 2.40–2.33 (t, *J =* 13.0 H_Z_, 1H, 4-Hb). Positive ESI-MS *m*/*z*: 356.2 [M + H]^+^. HR-MS for C_21_H_25_NO_4_ [M + H]^+^; calcd. 356.1856, found: 356.1864.

#### 3.1.16. Preparation of 11-Ethoxyisocorydine (**5c**)

A mixture of isocorydine (200 mg, 0.59 mmol), Tetrabutylammonium bromide (16 mg, 0.03 mmol), and K_2_CO_3_ (163 mg, 1.18 mmol) in anhydrous THF (10 mL) was successively added CH_3_CH_2_I (203 mg, 1.30 mmol) over a 10-min period and stirred for 8 h at room temperature. The reaction mixture was condensed and extracted with CHCl_3_. The combined extracts were dried over anhydrous Na_2_SO_4_, filtered, and concentrated to get a crude product, which was purified by silica gel column chromatography (CH_2_Cl_2_/MeOH 50:1), yielding compound **5c** (80 mg, 23%) as a colorless oil. ^1^H-NMR (400 MH_Z_, CDCl_3_, δ; ppm) 6.95 (d, *J =* 8.0 H_Z_, 1H, 8-H), 6.83 (d, *J =* 8.0 H_Z_,1H, 9-H), 6.66 (s, 1H, 3-H), 3.87 (d, *J =* 2.8 H_Z_, 6H, 1-OCH_3_, 2-OCH_3_), 3.84–3.79 (m, 1H, 6a-H), 3.66 (s, 3H, 10-OCH_3_), 3.19–3.14 (m, 1H, 7-Ha), 3.05–2.98 (m, 2H,11-CH_2_), 2.87–2.83 (m, 1H, 7-Hb), 2.71–2.67 (m 1H, 5-Ha), 2.54 (s, 3H, -NCH_3_), 2.50–2.47 (m, 4-Ha), 2.40–2.34 (t, 1H, *J =* 12.8 H_Z_, 4-Hb), 1.14–1.10 (t, *J =* 7.0 H_Z_, 3H, 11-CH_3_). ^13^C-NMR (100 MH_Z_, CDCl_3_, δ; ppm) 15.79 (q, 11-CH_2_CH_3_), 25.87 (t, C-7), 33.34 (t, C-4). 56.21 (q, 10-OCH_3_), 60.96 (q, 1-OCH_3_), 61.14 (q, 2-OCH_3_), 68.89 (t, 11-CH_2_CH_3_), 111.41 (d, C-3), 112.72 (d, C-9), 122.04 (d, C-8), 124.00 (C-7a), 125.01 (s, C-1a), 127.00 (s, C-1b), 146 .89 (s, C-1), 147.04 (s, C-10), 152.82 (s, C-11), 153.42 (s, C-2). Positive ESI-MS *m*/*z*: 370.2 [M + H]^+^. HR-MS for C_22_H_27_NO_4_ [M + H]^+^; calcd. 370.2013, found: 370.2022.

#### 3.1.17. Preparation of *N*-Methylisocorydine (**5d**) 

In a similar manner used in the preparation of **2d**, isocorydine (200 mg, 0.59 mmol) was subjected to a *N*-methylation reaction to give compound **5d** (183 mg, 88% yield) as a white solid. ^1^H-NMR (400 MH_Z_, CDCl_3_, δ; ppm) 8.60 (s, 1H, 11-OH), 7.01 (d, *J =* 8.0 H_Z_, 1H, 8-H), 6.89 (d, *J =* 8.0 Hz, 2H, 9-H), 6.86 (s, 1H, 3-H), 4.77–4.73 (dd, *J =* 12.0 H_Z_, *J =* 5.2 H_Z_, 1H, 6a-H), 4.24 (d, *J =* 13.2, 1H, 5a-H), 3.96 (s, 3H, 1-OCH_3_), 3.89 (s, 3H, 2-OCH_3_), 3.76 (s, 3H, 10-OCH_3_), 3.74 (s, 3H, -NCH_3_), 3.65–3.58 (m, 2H, 7a-H), 3.54–3.41 (m, 2H, 5b-H, 7a-H), 3.33 (s, 3H, -NCH_3_), 3.11–3.07 (m, 1H, 4-Ha), 2.88–2.81 (t, *J =* 12.0 H_Z_, 1H, 4-Hb). Positive ESI-MS *m*/*z*: 356.2 [M]^+^.

#### 3.1.18. Preparation of (*Z*)-*N*-2,3-Dimethoxybenzylidene-3,4-Dimethoxy-2-Phenyl-Ethanamine (**6a**)

A mixture of 2-(3,4-dimethoxy-phenyl)-ethylamine (1 g, 5.5 mmol) and 2,3-dimethoxy-benzoic acid (1.1 g, 6.1 mmol) in methylbenzene (50 mL) was heated to 120 °C for 8 h. The reaction mixture was condensed and extracted with EtOAC. The combined extracts were washed with water three times, dried over anhydrous Na_2_SO_4_, filtered, and concentrated. The crude product was drawn off by a vacuum pump over 4 h to give compound **6a** (1.78 g, 92% Purity, 91% yield) as a brown-red oil. ^1^H-NMR (400 MH_Z_, CDCl_3_, δ; ppm) 7.54 (d, *J =* 7.6 H_Z_ 1H, N-CH_2_CH_2_Ph-2H), 7.09–7.05 (t, *J =* 8.0 H_Z_, 1H, N-CH_2_Ph-4H), 6.96 (d, *J =* 8.0 H_Z_, 1H, N-CH_2_CH_2_Ph-5H), 6.76 (d, *J =* 12.0 H_Z_, 3H, N-CH_2_CH_2_Ph-6H, N-CH_2_Ph-3H, N-CH_2_Ph-5H), 3.90–3.88 (m, 1H, N-CH_2β_), 3.87–3.76 (m, 12H, 4×-OCH_3_), 2.98–2.94 (t, *J =* 7.0 H_Z_, 2H, N-CH_2α_). Positive ESI-MS *m*/*z*: 330.2 [M + H]^+^.

#### 3.1.19. Preparation of *N*-2,3-Dimethoxybenzyl-3,4-Dimethoxy-2-Phenylethanamine (**6b**)

Amixture of **6a** (1.5 g, 4.6 mmol) and Pd/C(5%, 150 mg) in MeOH (50 mL) was stirred under a hydrogen atmosphere (1 bar) for 8 h. The MeOH-insoluble material was filtered off and the filtrate was evaporated to give a crude product, which was purified by silica gel column chromatography (CH_2_Cl_2_/petroleum ether 1:1), yielding compound **6b** (1.3 g, 87%) as a yellow oil. ^1^H-NMR (400 MH_Z_, CDCl_3_, δ; ppm), 7.01–6.97 (t, *J =* 8.0 H_Z_, 1H, N-CH_2_Ph-4H), 6.83 (d, *J =* 6.4 H_Z_, 2H, N-CH_2_CH_2_Ph-5H, N-CH_2_CH_2_Ph-6H), 6.78 (d, *J =* 8.0 H_Z_, 1H, N-CH_2_CH_2_Ph-5H), 6.73 (d, 2H, *J =* 11.6, N-CH_2_Ph-3H, N-CH_2_Ph-5H), 3.84–3.77 (m, 12H, 4×-OCH_3_), 2.87–2.83 (t, *J =* 7.0 H_Z_, 2H, N-CH_2α_), 2.78–2.74 (t, *J =* 6.8 H_Z_, 2H, N-CH_2β_), 1.89 (s, 2H, N-CH_2_). Positive ESI-MS *m*/*z*: 310.2 [M + H]^+^.

#### 3.1.20. Preparation of *N*-Methyl-*N*-2,3-Dimethoxybenzyl-3,4-Dimethoxy-2-Phenyl-Ethanamine (**6c**)

To a mixture of **6b** (1.2 g, 3.6 mmol) and K_2_CO_3_ (1.0 g, 7.2 mmol) in anhydrous THF (20 mL),CHI (610.6 mg, 4.3 mmol) was slowly added under an ice-bath over a 10-min period. The solution was stirred for 4 h at room temperature, condensed, and extracted with EtOAC. The combined extracts were dried over anhydrous Na_2_SO_4_ and filtered, and the filtrate was concentrated to give a crude product, which was purified by silica gel column chromatography (CH_2_Cl_2_/petroleum ether 10:1), yielding compound **6c** (950 mg, 76% yield) as a colorless oil. ^1^H-NMR (400 MH_Z_, CDCl_3_, δ; ppm) 7.06–7.00 (dd, 2H, *J =* 9.6 H_Z_, *J =* 4.8 H_Z,_ N-CH_2_CH_2_Ph-5H, N-CH_2_CH_2_Ph-6H), 6.86 (t, 1H, *J =* 4.2 HZ, N-CH_2_Ph-4H), 6.78 (d, 1H, *J =* 8.4, N-CH_2_CH_2_Ph-2H), 6.73 (d, 2H, *J =* 7.2, N-CH_2_Ph-5H, N-CH_2_Ph-3H), 3.86–3.82 (m, 12H, 4×-OCH_3_), 3.74 (s, 2H, N-CH_2_), 2.87 (t, 2H, *J =* 7.2, N-CH_2α_), 2.77–2.71 (m, 2H, N-CH_2β_), 2.37 (s, 3H). Positive ESI-MS *m*/*z*: 346.2 [M + H]^+^.

### 3.2. Pharmacological Evaluation

#### 3.2.1. Materials

Verapamil hydrochloride (Shanghai Harvest Pharmaceutical Co., Ltd., Shanghai, China), lidocaine hydrochloride (Shanghai ZhaohuiPharmaceutical Co., Ltd., Shanghai, China), isocorydine, crebanine, and stephanine were used as positive control groups. Normal saline (NS) (Kunming Nanjiang Pharmaceutical Co., Ltd., Kunming, China) was used as negative control group. Other chemicals used—chloral hydrate, barium chloride, chloroform, sodium hydroxide, and hydrochloric acid were of analytical grade.

All aporphine alkaloid derivatives were used for the tested drug groups, and were synthesized, purified, and determined according to Section 3.1.2, Section 3.1.3, Section 3.1.4, Section 3.1.5, Section 3.1.6, Section 3.1.7, Section 3.1.8, Section 3.1.9, Section 3.1.10, Section 3.1.11, Section 3.1.12, Section 3.1.13, Section 3.1.14, Section 3.1.15, Section 3.1.16, Section 3.1.17, Section 3.1.18, Section 3.1.19 and 3.1.20 in this paper. The compounds were dissolved in 10% hydrochloric acid solution and were adjusted to pH5–7 with 40% sodium hydroxide.

#### 3.2.2. Animals

The experiments were carried out on Kunming mice (28–35 g) and SD rats (180–210 g), which were provided by Hunan SJA Laboratory Animal Co., Ltd. (SYXK (Dian) K 2011-0011, Changsha, China). Half of the animals were male and half were female, and they were housed in a controlled environment (temperature 22 ± 1 °C; 60% ± 10% humidity; in a 12/12 h light-dark cycle) with free access to standard pellet artificial diet and tap water. All animal experiments performed were in compliance with the Animal Experimental Care and Ethic Committee of Yunnan University of Traditional Chinese Medicine.

Determination of the dosage for tested drugs: The animals were first given 5 mg/kg of tested drugs, based on the effective dose of crebanine in mice and rat [7]. The dosage was reduced or increased according to the exhibited symptoms of poisoning, or death, and the antiarrhythmic effect on the animals.

#### 3.2.3. Statistical Analyses

Data are expressed as the means ± SD and incidence ratio. Statistical significance was calculated using the Chi-square test. Differences were considered significant when *p <* 0.05. LD_50_ was determined using a related formulate of up-and-down procedure (UDP) [28].

#### 3.2.4. Antiarrhythmic Activity Test of Aporphine Derivatives in a Mouse Model of Ventricular Fibrillation Induced by CHCl_3_

Mice were randomly divided into a negative control group (NS), 4 positive control groups (verapamil hydrochloride, crebanine, iscorydine, and stephanine), and 19 tested groups for aporphine derivatives (Figure 1). Tested drugs were injected though the caudal vein at a chosen dose. Five minutes later, each mouse was placed in a 500-mL inverted beaker, which contained a cotton ball that had been soaked with 1mL of chloroform, and then were removed immediately after respiratory arrest. The chest of the asphyxiated mouse was opened in order to observe the condition of ventricular fibrillation (VF) [7,29]. The occurrence ratios of VF in mice are listed in Figure 1.

#### 3.2.5. Anti-Arrhythmic Activity of Five Kinds of Aporphine Derivatives in a Rat Model of Arrhythmia Induced by BaCl_2_

Rats were randomly divided into 10 groups: an NS group, 4 groups of positive control (lidocaine, crebanine, iscorydine and stephanine) groups, and 5 tested groups of active derivatives (**2a**–**d** and **4a**) (Table 1). First, rats were anesthetized with 10% choral hydrate (ip, 300 mg/kg), fixed on their backs and connected to a physiological signal acquisition system in order to record normal II lead electrocardiogram (ECG). Then, a barium chloride solution was injected into the caudal vein of each rat (4 mg/kg, at a volume of 1 mL/kg) to trigger arrhythmia. Three minutes later, rats were injected with different treatments though the lingual vein at a certain doses. Then, ECG was recorded. The number of rats that recovered to a normal sinus rhythm, the duration needed to recover, and number of rats that maintained normal rhythm for more than 3 min, 5 min, and 20 min, in each group, were calculated [7,29] (Table 1).

#### 3.2.6. Acute Toxicity Test in Mice

Mice were injected with the test compounds via the caudal vein at a certain dose. The D_m_ (100% death) and D_n_ (0% death) of the compounds were found in preliminary tests; then, 5–6 dose groups (*n* = 10) of the compounds were set up, according to the requirements of the up-and-down procedure (UDP) method, death number was recorded and LD_50_ was determined [28].

## 4. Conclusions

In this study, derivatives of three kinds of aporphine alkaloids, crebanine, isocorydine, and stephanine, were designed and synthesized through the reactions of ring-opening, *N*-substitution, demethylation, methylation, acetylation, quaternization, and dehydrogenation. The use of NBS for the bromination reaction was successfully applied to the synthesis of the three kinds of bromocrebanines. Among these derivatives, compound **2b**, dibromocrebanine, proved to be the most promising candidate, showing maximum potency against arrhythmia and minimum toxicity compared to the other compounds prepared in this family. Derivatives **2a**, **2c**, **2d**, and **4a** also displayed impressive potency. Finally, derivative **1d** exhibited a lower, though still significant, activity.

The relationship of the structure–antiarrhythmic activities of aporphines are summarized as: (1) C-1,C-2-methylenedioxy group on ring A of aporphines increases the planar configuration of molecules and plays an important role in both activity and toxicity; (2) a closed structure of the B ring is essential for both antiarrhythmic efficacy and toxicity; additionally, *N*-quaternization of ring B could increase toxicity; (3) levoduction of **6a** in ring C is an active group; and (4) 8-, 9-, and 10-methoxy groups on ring D is an important functional groups. Further explorations are underway to evaluate the biological activities and the ion channel mechanisms of these aporphine derivatives.

## Figures and Tables

**Table 1:** Antiarrhythmic activity of fiveaporphine derivatives (**2a**–**d** and **4a**) in the model of arrhythmia, induced by BaCl_2_ in anesthetized SD rats (iv) (*n* = 10).

Compounds	Dose (mg/kg)	Numbers of Rat Recovering	Recovery Time (s)	Numbers of Rat Maintaining Time ≥ 3 min	Numbers of Rat Maintaining Time ≥ 5 min	Numbers of Rat Maintaining Time ≥ 20 min
NS	--	0	--	0	0	0
Lidocaine	5	7 **	22.2 ± 8.0	6 **	2	1
Crebanine	5	8 **	30.3 ± 31.8	5 **	3	3
Stephanine	5	7 **	35.0 ± 8.1	6 **	4 *	3
Isocorydine	10	9 **	736.4 ± 489.1	8 **	8 **	4 *
3,10,11-Tribromocrebanine (**2a**)	15	7 **	125.5 ± 145.9	2	1	0
10,11-Dibromocrebanine (**2b**)	7.5	8 **	113.5 ± 136.8	8 **	7 **	5 **
3-Bromocrebanine (**2c**)	7.5	8 **	73.1 ± 58.2	4 *	2	2
*N*-methylcrebanine (**2d**)	3	8 **	131.9 ± 122.9	5 **	5 **	2
Dehydrostephanine (**4a**)	5	10 **	23.0 ± 11.0	0	0	0

The chi-square test was used to estimate the significance between the numbers of rat recovering and the numbers of rat maintaining time ≥3 min, 5 min, and 20 min in all comparisons. Significantly different from the control group, * *p <* 0.05, ** *p <* 0.01. Values of recovery time are expressed as mean ± SD. SD represents standard deviation.

**Table 2:** Acute toxicity tests of three crebanine derivatives (**2b**, **2c**, and **2d**) in mice (*n* = 10).

Compounds	LD_50_ (mg/kg)	95% Confidence Intervals (CIs)
10,11-Dibromocrebanine (**2b**)	59.62	62.23, 57.01
3-Bromocrebanine (**2c**)	65.15	66.28, 64.02
*N*-methylcrebanine (**2d**)	6.43	7.53, 5.34
Crebanine [14]	9.38	8.24, 10.53

**Table 3:** *O*-Demethylation reaction of crebanine.

Entry	Solvent	Temperature (°C)	Time (h)	Product	Yield (%)
1	48%HBr	120	3.5	--	--
2	48%HBr/CH_3_COOH	130	4	**1d**	70
3	HI	100	2	--	--
4	BBr_3_/CH_2_Cl_2_	25	3	--	--
5	BCl_3_/CH_2_Cl_2_	25	12	**2d**	10
6	AlBr_3_/CH_2_Cl_2_	25	12	**2d**	10
7	AlBr_3_/PhNO_2_	40	4	**2d**	20

“--” represents that no desired product was obtained.
